# Tuning Interface Bridging Between MoSe_2_ and Three-Dimensional Carbon Framework by Incorporation of MoC Intermediate to Boost Lithium Storage Capability

**DOI:** 10.1007/s40820-020-00511-4

**Published:** 2020-08-25

**Authors:** Jing Chen, Yilin Luo, Wenchao Zhang, Yu Qiao, Xinxin Cao, Xuefang Xie, Haoshen Zhou, Anqiang Pan, Shuquan Liang

**Affiliations:** 1 School of Materials Science and Engineering, Central South University, Changsha, 410083 Hunan People’s Republic of China; 2 Energy Interface Technology Group, National Institute of Advanced Industrial Science and Technology, 1-1-1, Umezono, Tsukuba, 305-8568 Japan; 3 Institute for Superconducting and Electronic Materials, School of Mechanical, Materials, Mechatronics and Biomedical Engineering, Faculty of Engineering and Information Sciences, University of Wollongong, Wollongong, NSW 2500 Australia; 4 Key Laboratory of Advanced Energy Materials Chemistry (Ministry of Education), Nankai University, Tianjin, 300071 People’s Republic of China

**Keywords:** Interface engineering, Porous carbon framework, MoSe_2_ nanodots, MoC, Heterostructure, Battery

## Abstract

**Highlights:**

- MoSe_2_/MoC/C multiphase boundaries boost ionic transfer kinetics.
- MoSe_2_ (5–10 nm) with rich edge sites is uniformly coated in N-doped framework.
- The obtained MoSe_2_ nanodots achieved ultralong cycle performance in LIBs and high capacity retention in full cell.

**Abstract:**

Interface engineering has been widely explored to improve the electrochemical performances of composite electrodes, which governs the interface charge transfer, electron transportation, and structural stability. Herein, MoC is incorporated into MoSe_2_/C composite as an intermediate phase to alter the bridging between MoSe_2_- and nitrogen-doped three-dimensional (3D) carbon framework as MoSe_2_/MoC/N–C connection, which greatly improve the structural stability, electronic conductivity, and interfacial charge transfer. Moreover, the incorporation of MoC into the composites inhibits the overgrowth of MoSe_2_ nanosheets on the 3D carbon framework, producing much smaller MoSe_2_ nanodots. The obtained MoSe_2_ nanodots with fewer layers, rich edge sites, and heteroatom doping ensure the good kinetics to promote pseudo-capacitance contributions. Employing as anode material for lithium-ion batteries, it shows ultralong cycle life (with 90% capacity retention after 5000 cycles at 2 A g^−1^) and excellent rate capability. Moreover, the constructed LiFePO_4_//MoSe_2_/MoC/N–C full cell exhibits over 86% capacity retention at 2 A g^−1^ after 300 cycles. The results demonstrate the effectiveness of the interface engineering by incorporation of MoC as interface bridging intermediate to boost the lithium storage capability, which can be extended as a potential general strategy for the interface engineering of composite materials.
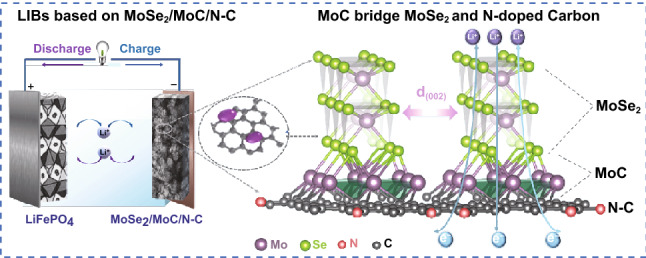

**Electronic supplementary material:**

The online version of this article (10.1007/s40820-020-00511-4) contains supplementary material, which is available to authorized users.

## Introduction

Transition metal dichalcogenide (TMD) materials MX_2_ (M = transition metal; X = chalcogen) with lamellar structure have received broad attentions in the fields of batteries, supercapacitors, catalysts, and sensors due to their large layer distance and high surface area [1–6]. Among them, transition metal selenides (MSe_2_) have higher conductivity than the corresponding sulfides (MS_2_) and oxides (MO_2_). Moreover, the bond strength of M–Se is weaker than M–S or M–O, which is kinetically favorable for conversion reactions [7, 8]. Specifically, molybdenum diselenide (MoSe_2_) possesses large interlayer distance (0.65 nm, two times larger than that of commercial graphite) because of weak van der Waals forces and relatively high electronic conductivity (1 × 10^−3^ S m^−1^) triggered from the narrow band gap (1.1 eV) [9–11], making it a promising electrode material for lithium-ion batteries (LIBs). However, MoSe_2_ electrode still suffers from large volume variations, inherently low conductivity, and adverse reaction during cycling, which leads to severe capacity fading and inferior rate performance [8, 12–15].

To address aforementioned concerns, great efforts have been focused on nano-/microstructure design and carbon modification. In general, nanosized materials can effectively eliminate the effect of volume variations, providing a short ion diffusion length and offer large pseudo-capacitance, which lead to fast ionic migration and superior rate performance [16–19]. However, the large contact area between electrode materials and electrolyte may increase the side reaction and cause the dissolution of the electrode materials. Another effective strategy is making TMD and carbon composites, in which carbon serves as a soft matrix to buffer the volume variations and fast electron conductor [20–23]. Particularly, three-dimensional (3D) porous carbon skeletons are preferred because of their extra capability to tolerant the volume changes. Zhao et al. have achieved high-performance Co_3_O_4_ lithium-ion battery anode by employing 3D carbon network substrate [24]. Cao and co-workers synthesized 3D porous Mo_2_C/C architecture to improve the lithium storage capacity [25]. Although the electrochemical performances are improved greatly, the long-term cycling stability is rear reported. The difficult can be attributed to the weak bonding between the active material and carbon substrates, or the mismatch of their volume variations, which degrade the structural stability of the electrode upon long-term cycling.

Interface engineering can introduce distortions, dislocations, and lattice defects, with distinguished electronic structures, which are long-range disorder and, hence, can lower the activation barrier, thus boosting reaction kinetics [5, 26, 27]. More recently, through interface coupling by chemical bonds, previous theoretical calculation and practical experiments both have revealed that Mo–O–C or Mo–C bonds between carbon and MoSe_2_ can effectively enhance electronic conductivity and structure stability through the interface [28, 29]. However, the portion of the chemical bonding between the active material and substrate is still too limited to stabilize structure. Therefore, it is highly expected to increase the chemical bonds at the interface or develop new strategy to improve the stability of composites, in particular for long-term cycling applications. Introducing intermediate material to bridge the active material and the substrate presents to be a wise choice. Comparing to MoSe_2_, MoC owns intrinsic higher electrical conductivity and chemical stability [30–32]. The structural stability and charge transportation will be greatly improved by the incorporation of MoC into the composite, which bridges MoSe_2_ and carbon substrates by MoSe_2_–MoC–carbon connection.

Herein, we reported the temperature-induced one-step incorporation of MoC as an intermediate phase to bridge MoSe_2_- and N-doped three-dimensional carbon framework, which essentially improve the interfacial structural stability of the composite. Moreover, the in situ formed MoC can effectively inhibit the overgrowth of MoSe_2_ nanocrystals and constrain MoSe_2_ to nanodots with few layers and rich edge sites. The obtained materials guarantee bi-continuous fast electron/ion transport and highly reversible conversion reactions and enable fast reaction kinetics and good structural stability. As expected, 3D porous MoSe_2_/MoC/N–C electrodes deliver much improved long-term cycling stability. It retains 90% of its initial capacity after 5000 cycles at 2 A g^−1^. And the assembled LiFePO_4_//MoSe_2_/MoC/N–C full cell also exhibits a high reversible capacity and good cyclic stability (86% capacity retention after 300 cycles at 2 A g^−1^).

## Experimental

### Synthesis of 3D Porous MoSe_2_/MoC@N–C and MoSe_2_/C

All chemicals were used as received without further purification. 0.4 g of (NH_4_)_6_Mo_7_O_24_·4H_2_O, 0.4 g of PVP and 1.2 g of NaCl were dissolved in 30 mL of distilled water. After stirring for 1 h at room temperature, the resulting transparent solution was quick-frozen with liquid nitrogen and further freeze-dried for 30 h at − 40 °C in vacuum to yield a precursor. Then, the precursor was mixed with selenium powders in a mass ratio of 5:1 and annealed under Ar atmosphere at 600 or 800 °C for 3 h with a heating rate of 5 °C min^−1^. After that, 3D porous MoSe_2_/C and MoSe_2_/MoC@N–C were obtained after dissolving NaCl in deionized water. For comparison, bare MoSe_2_ was prepared by annealing a mixture of ammonium molybdate tetrahydrate and selenium powders at 800 °C for 3 h with a heating rate of 5 °C min^−1^ under Ar atmosphere. MoC/C could be obtained by annealing the same precursor under Ar atmosphere at 750 °C for 3 h with a heating rate of 5 °C min^−1^ without Se powder.

### Characterizations

The structure features of the samples were performed by X-ray diffraction (XRD, Rigaku D/max 2500), Raman microscope (Horiba Jobin–Yvon, Lab Ram Aramis), and X-ray photoelectron spectroscopy (XPS, AXIS-ULTRA DLD-600 W system). The morphologies and energy-dispersive X-ray (EDX) element mapping (SEM, Quanta FEG 250) were characterized by scanning electron microscopy and Transmission electron microscope (TEM, JEOL JEM-2100F). Specific surface areas were calculated by the multipoint Brunauer–Emmett–Teller (BET) method.

### Electrode Fabrication and Electrochemical Measurement

Electrochemical performances were evaluated by using 2032-type coin half cells with metallic lithium served as the anode. Working electrodes were prepared by casting a slurry containing the active material (70 wt%), Super P (15 wt%) and sodium carboxymethyl cellulose (15 wt%) dispersed in distilled water on a clean Cu foil current collector. Each electrode in our experiment has an area of 1.1304 cm^−2^, and the loading of active material for each electrode was about 1.0–1.2 mg. All cells were assembled in a glove box (Mbraun, Germany) filled with ultrahigh-purity argon. For LIB assembly, polypropylene membrane and 1 M LiPF_6_ in ethylene carbonate (EC)–dimethyl carbonate (DMC)–diethyl carbonate (DEC) (1:1:1 in volume) were used as a separator and electrolyte, respectively. As for the assembly of full cell, a pre-lithiation procedure was carried on to compensate the loss of lithium during the initial cycle in half cell. To ensure the maximized material utilization and reasonably evaluate the electrochemical property of the MoSe_2_/MoC@N–C, the full cell in this work was assembled based on the capacity ratio of ≈ 1.2:1 between the LiFePO_4_ cathode and MoSe_2_/MoC/N–C anode.

The cyclic voltammetry (CV) measurements were taken on an electrochemical workstation (CHI660C) at a scan rate of 0.1 mV S^−1^ in the voltage range of 0.01–3 V (vs. Li^+^/Li). The electrochemical impedance spectrometry (EIS) data were collected on a ZAHNER-IM6ex electrochemical workstation (ZAHNER Co., Germany) in the frequency range of 100 kHz to 10 mHz. Land battery tester (Land CT 2001A, Wuhan, China) was employed to investigate the galvanostatic charge/discharge performances, and all tests were conducted at room temperature.

## Results and Discussion

### Formation Mechanism and Structure Characterization of Materials

**Scheme 1:**
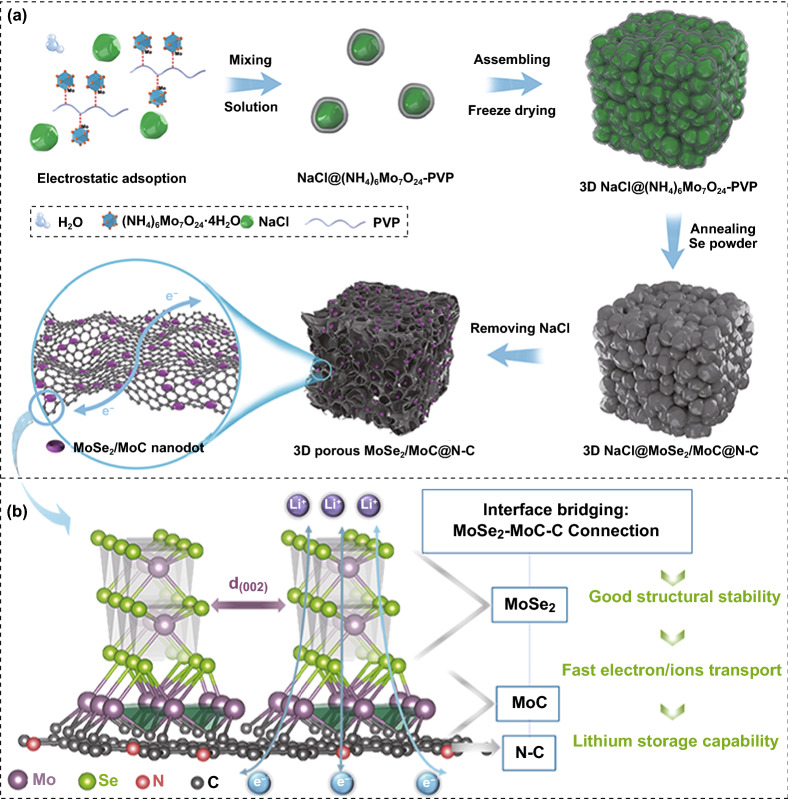
Schematic illustration of **a** the formation process of interconnected 3D porous structure of obtained MoSe_2_/MoC/N–C nanocomposite and **b** model of interface bridging between MoSe_2_- and N-doped carbon network

The synthetic mechanism of the 3D porous MoSe_2_/MoC/N–C and advantages of the structure are shown in Scheme 1. As a nitrogen-rich carbon network source, polyvinyl pyrrolidone (PVP) is a nonionic surfactant that can adsorb molybdate ions to facilitate the in situ formation of Mo–C bond. When the precursor annealed at high temperature, molybdenum can react with carbon to form MoC. Selenium powder is used to form MoSe_2_. And NaCl is employed as a template to create pores due to its environmental friendliness, cheapness, and high melting point. The obtained porous structure with 3D electrically conductive pathways possesses both large surface areas and abundant mass transportation channels, guaranteeing the good contact between electrolyte and electrode interface. More importantly, as shown in Scheme 1b, different from previous reports of MoSe_2_/C composites, MoSe_2_ and carbon substrate were bridged by highly conductive MoC interphase, forming a tri-phase MoC/N–C heterostructure where the MoSe_2_/MoC/N–C connection can boost fast charge kinetics and highly reversible conversion, leading to improved long-term cycling stability. This MoSe_2_/MoC/N–C connection also induced the growth of MoSe_2_ to terrace-terminated mode (illustrated in Scheme S1), resulting few-layered MoSe_2_ nanodots, and thus, greatly contributed to pseudo-capacitance effect and guaranteed the fast the ion diffusion kinetics, which synergistically boosts lithium storage capability.

**Fig. 1:**
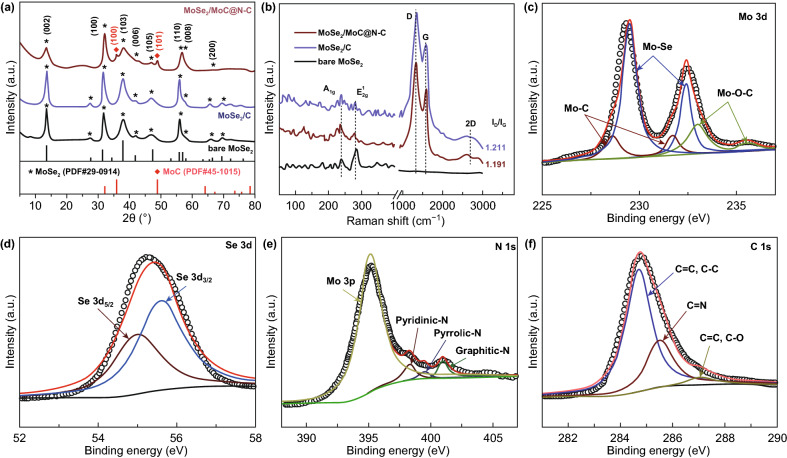
**a** XRD patterns and **b** Raman spectrum of bare MoSe_2_, MoSe_2_/C, and MoSe_2_/MoC/N–C; XPS spectra of MoSe_2_/MoC/N–C: **c** Mo 3d, **d** Se 3d, **e** N 1 s, and **f** C 1 s

**Fig. 2:**
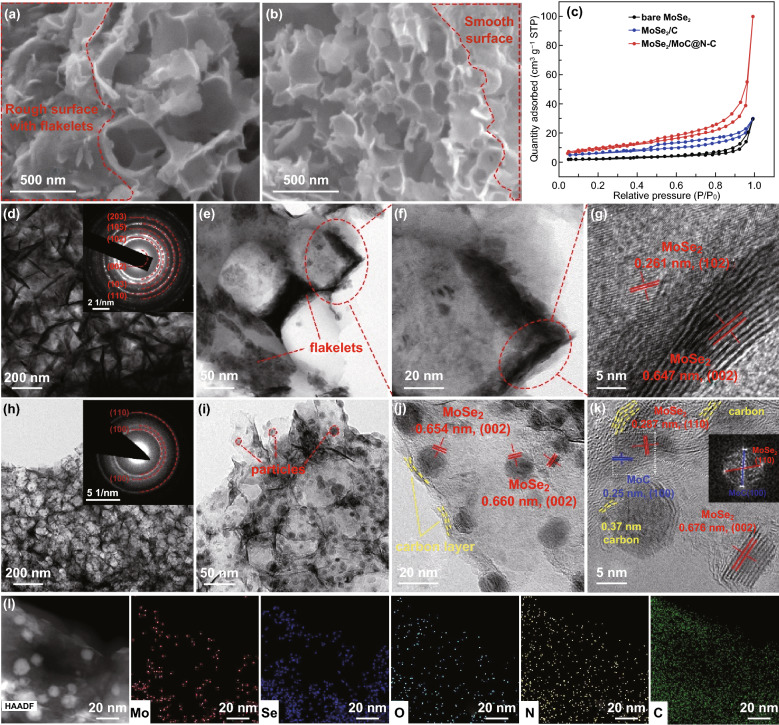
SEM images of **a** MoSe_2_/C and **b** MoSe_2_/MoC/N–C; **c** statistical data of surface area; **d**–**f** TEM and **g** HRTEM images of MoSe_2_/C; **h**–**j** TEM and **k** HRTEM images of MoSe_2_/MoC/N–C (inset: FFT of the selected area); **l** elemental mappings of MoSe_2_/MoC/N–C

More specific characterizations of material are comprehensively presented within Figs. 1 and 2. As shown in Fig. 1a (XRD patterns), the as-prepared sample annealed under 800 °C exhibited two additional diffraction peaks located at 35.7° and 48.7° compared to MoSe_2_ phase (JCPDS No. 29-0914, 2H-MoSe_2_), which are corresponding to MoC phase (JCPDS No. 45-1015). Specifically, compared to bare MoSe_2_ and MoSe_2_/C, the broadened diffraction peaks in MoSe_2_/MoC/N–C sample indicate its relatively small crystal size. And the weaker intensity of (002) peak indicates rich defects along [002] direction [28]. Unlike the crystallinity normally increase with the rising of the annealing temperature (800 °C), the intensity and crystallites of MoSe_2_ in the composite decreased. The XRD patterns suggest the growth of MoSe_2_ may be changed. No detection of XRD peaks for Mo metal indicates the easier formation of MoC and its stable state at 800 °C.

As for the Raman characterization, all the samples present two bands at around 240 and 280 cm^−1^ (Fig. 1b), which are assigned to the A_g_^1^ and E_2g_^1^ stretching of MoSe_2_, respectively. The A_g_^1^-related peak is attributed to the exclusive peak of MoSe_2_ 2H structure, preferred for edge-terminated mode, while the E_2g_^1^ peak is assigned to the characteristic peaks of MoSe_2_ 1T structure, favored for terrace-terminated MoSe_2_ [13, 33, 34]. Thus, 1T/2H phases of MoSe_2_ were consisted in three synthesized samples. Moreover, the increased intensity ratio of A_g_^1^/E_2g_^1^ from bare MoSe_2_ to MoSe_2_/MoC/N–C suggests the decreased thickness and increased exposed edge sites of MoSe_2_ in the interconnected 3D porous structure. The D and G bands locating at 1375 and 1590 cm^−1^ confirm the presence of disordered and graphitic carbon structures within interconnected 3D porous superstructure, respectively [12, 35, 36]. The content of carbon was calculated as 34.6% in the MoSe_2_/C composite, determined by thermal gravimetric (TG) analysis as shown in Fig. S1, and the residual product (MoO_3_) is verified by the XRD pattern in the inset. Furthermore, the XPS spectrum shows the existence of Mo, Se, C, and N elements (Fig. S2). The Mo 3d XPS spectrum (Fig. 1c) shows three doublets: 229.5 and 232.4 eV assigned to Mo–Se bonds, 233.3 and 235.7 eV corresponding to Mo–O–C, and 228.6 and 231.7 eV corresponding to Mo–C bonding [13, 24, 33, 37], confirming the formation of MoC in the MoSe_2_/MoC/N–C nanocomposite, which is consistent with the XRD results. More importantly, the formation of Mo–O–C and Mo–C bonds could be demonstrated, which implies the strong electronic coupling at the interface between MoC, MoSe_2_and 3D porous carbon. The formation of Mo–O–C bonds was mainly due to the combination of Mo ion with the carbonyl group (–C=O) in the PVP. Previous experiments and theoretical calculations have shown that Mo–C or Mo–O–C bonds in MoSe_2_/C nanocomposite could facilitate the ion and electron transportation in the composite [28, 29]. For the Se 3d XPS spectra, two typical peaks at 55.5 and 54.8 eV correspond to the 3d_3/2_ and 3d_5/2_ peaks of Se^2−^ in the MoSe_2_/MoC/N–C nanocomposite (Fig. 1d). The C 1*s* profile in Fig. 1f can be deconvoluted into three individual peaks at 285.5 eV (C–N), 284.7 eV (C–C and C=C), and 287.1 eV (C–O, C=O). The formation of C–N bonds demonstrated the nitrogen doping in the carbon substrate. Furthermore, the N 1*s* peaks are partially overlapped with Mo 3*p* peaks (at 395.1 eV) and could be deconvoluted as graphitic N at 400.9 eV, pyrrolic-N at 399.5 eV, and pyridinic-N at 398.3 eV [13, 24, 33, 37]. The nitrogen doping could introduce more defects and electroactive sites, resulting in good electron transportation in the composite [38].

As depicted in Fig. S3, pure MoSe_2_ agglomerates into irregular bulks about 20 μm in width. However, as expected, both MoSe_2_/C and MoSe_2_/MoC/N–C possessed a highly interconnected three-dimensional porous network with microstructure (Fig. 2). Interestingly, compared to MoSe_2_/C, MoSe_2_/MoC/N–C possesses more uniform pore distribution and thinner network thickness (Fig. 2a, b), which can be further confirmed by TEM observations. The uniform pores (about 80 nm) were created by dissolution of NaCl and the release of gases generated during the selenization. As a result, MoSe_2_/MoC/N–C composite possesses a large Brunauer–Emmett–Teller specific surface area (*S*_BET_) of 34.9 m^2^ g^−1^ and MoSe_2_@N–C possesses specific surface area of 21.5 m^2^ g^−1^, both of which are much larger than 7.9 m^2^ g^−1^ of bare MoSe_2_ (Fig. 2c). The detailed information of pore size distribution of three samples is shown in Fig. S4. The high *S*_BET_ and the porous structure are favorable to ion diffusion and structural stability upon cycling.

The semitransparent TEM images further indicate the ultrathin network structure of composites (Fig. 2d–k). When we zoom in, for MoSe_2_/C sample, thick nanosheets with various sizes were attached to or embedded in the network (Fig. 2e, f), which were proved as MoSe_2_ (Fig. 2g). In general, agglomeration is more likely to occur in high-temperature reactions. However, MoSe_2_ crystals in MoSe_2_/MoC/N–C sample (800 °C) are smaller and more dispersed than those in MoSe_2_/N–C (600 °C). In comparison, for MoSe_2_/MoC/N–C, the interconnected 3D porous architecture (Fig. 2h) is more obvious than that of MoSe_2_/C. Moreover, a large number of small particles with size of 5–20 nm are uniformly distributed in smooth N–C network (Fig. 2i, j). These small sizes are favorable to the lithium-ion intercalation/deintercalation. Figure 2j further indicates that the thin carbon layer totally coats the small MoSe_2_/MoC nanocrystal. The N–C capsule can effectively protect nanosized MoSe_2_ from adverse reactions and facilitate fast electron transport.

The high-resolution TEM (HRTEM) image (Fig. 2g, k) reveals that nanodots in MoSe_2_/MoC/N–C nanocomposite is composed of only a few layers (about 10 layers), much less than that of MoSe_2_/C. Furthermore, the layer distance of MoSe_2_/MoC/N–C (0.676 nm) is also larger than that of MoSe_2_/C (0.647 nm), which facilitate fast Li-ion transportations. Notably, the interlayer distances of 0.25 nm marked in Fig. 2k is assigned to the distance spacing of (100) plane of MoC crystal, which is in close contact with MoSe_2_ crystals. And the corresponding fast Fourier transform (FFT) result (inset in Fig. 2k) revealed the (110) and (100) plane of the MoSe_2_ and MoC, respectively, further confirming the formation of MoSe_2_/MoC heterostructure. Because the carbon source of MoC is from the carbonized PVP (N–C substrate), the in situ formed MoC is bonded to carbon framework, which can be confirmed by Mo-C and Mo–O-C bonding from XPS (Fig. 1c). Therefore, it can be deduced that the intermediate MoC bridges N–C framework and MoSe_2_ through MoSe_2_/MoC/N–C connection, strengthening the interfacial coupling in tri-phase MoSe_2_/MoC/N–C superstructure, which enable fast charge transportation and good structural stability. And defects in MoSe_2_/MoC/N–C multiphase boundaries with distinguished electronic structures could lower the activation barrier, thus boosting reaction kinetics. In addition, around nanodots, some graphitic carbon fringes about 0.37 nm are observed, as indicated by the yellow dotted line (Fig. 2k). The graphitic carbon in the composite can provide conductive pathways for rapid electron transfer even at high current densities.

By comparing the structure of MoSe_2_/C and MoSe_2_/MoC/N–C composites (Fig. 2), it can be concluded that the in situ formed intermediate MoC could effectively inhibit the overgrowth of MoSe_2_ nanocrystals and expand their layer spacing. During the heating process, partial Mo reacted with Se to form MoSe_2_ first. When the reaction temperature rose to 800 °C, Mo and carbon reacted to form dispersion MoC, while MoSe_2_ crystals continue to nucleate and grow, forming MoSe_2_/MoC/C heterojunction. This MoSe_2_/MoC/C connection induced the growth of MoSe_2_ to terrace-terminated mode (illustrated in Scheme S1), resulting in few-layered and small-sized MoSe_2_ nanodots. Furthermore, the in situ formed MoC can pin heterostructure, preventing crystals form and agglomerating. The dark-field scanning TEM and the corresponding elemental mapping images of MoSe_2_/MoC/N–C reveal that Mo, Se, O, N, and C are evenly distributed on the ultrathin carbon wall (Figs. 2l and S12). The distribution of Mo and Se concentrate on the location of nanodots, indicating the formation of MoSe_2_ again, while the uniform distribution of N throughout the sample demonstrates that the 3D porous framework consists of N-doped carbon.

### Electrochemical Lithium-Ion Storage Performance and Reaction Kinetics

**Fig. 3:**
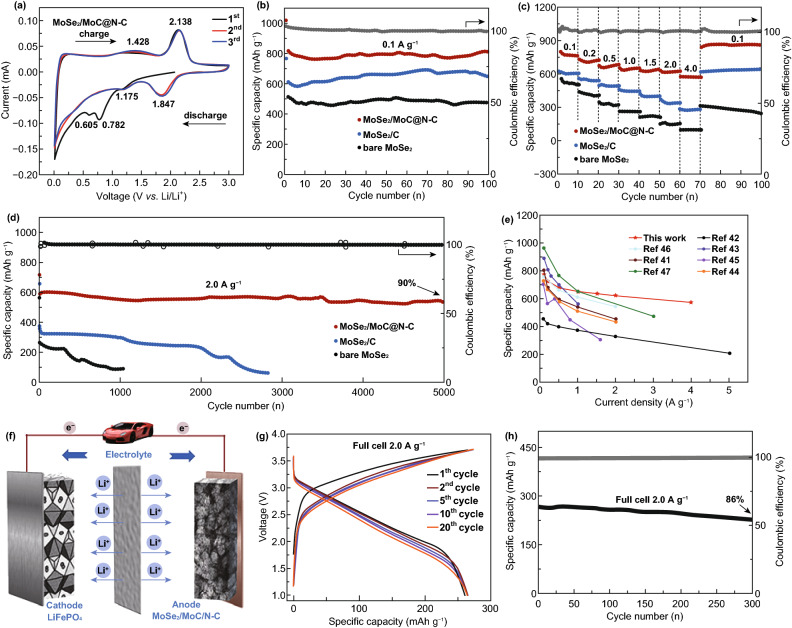
**a** Initial three cyclic voltammograms cycles of the MoSe_2_/MoC/N–C electrode at a scan rate of 0.1 mV s^−1^. **b** Cycling performance (0.1 A g^−1^), **c** rate performances, and **d** long-term cycling performances (2 A g^−1^) of bare MoSe_2_, MoSe_2_/C, and MoSe_2_/MoC/N–C electrodes between 0.01 and 3 V versus Li/Li^+^. **e** Comparison plot of rate performance between this work and previously published MoSe_2_/C composite-related works. **f** Schematic illustration of the LiFePO_4_//MoSe_2_/MoC/N–C full-cell configuration. **g** Charge/discharge profiles of the full cell. **h** Cycling performance of the full cell at 0.1 A g^−1^

The MoSe_2_/MoC/N–C composite was assembled to half and full cells to investigate their electrochemical performances. The cyclic voltammetry (CV) curves obtained during the initial 3 cycles (at a scanning rate of 0.1 mV s^−1^) exhibit multiple redox reactions for MoSe_2_/MoC/N–C electrode (Fig. 3a). During the first cathodic sweep, four obvious reduction peaks at 0.605, 0.782, 1.175, and 2.138 V can be clearly observed. The small peak at 1.175 V can be assigned to the reduction of MoC, and other peaks are well documented for MoSe_2_ [14, 31, 39]. These peaks are also consistent with the results in CV curves of MoSe_2_/C and MoC/C counterparts in Figs. S7b and S8c. The reduction peak at 0.782 V can be ascribed to the insertion of Li ion into MoSe_2_ to form Li_*x*_MoSe_2_, and the subsequent peak at 0.605 V can be ascribed to the reduction of Li_*x*_MoSe_2_ to Mo and Li_2_Se [28, 44]. In the first cycle, two oxidation peaks at 1.428 and 2.138 V correspond to a partial oxidation of Mo metal to form MoSe_2_ and oxidation of Li_2_Se to Se, respectively [14]. In the next cycles, reduction peaks at 0.605 and 0.782 V disappeared, but new peak at 1.847 V can be obtained for the conversion of Li_*x*_MoSe_2_ to Mo and Li_2_Se [41–44], while two oxidation peaks at 2.138 and 1.428 V remain constant. The shift of peaks of MoSe_2_ involved with the irreversible reaction [43–47]. However, the peak at 1.175 V corresponding to MoC remains constant (the same as this peak at CV curves of MoC/C in Fig. S8c), suggesting the reversible conversion reactions of MoC [31, 39]. Figure S6 presents charge/discharge profiles of selected cycles of MoSe_2_/MoC/N–C electrode; after initial cycle, two platforms at around 1.8 and 1.2 V can be ascribed to the contribution of MoSe_2_ and MoC in the composite electrode, respectively. The result is the same as peak voltages in CV curves. The initial charge and discharge capacities are 812 and 1014 mAh g^−1^, respectively, corresponding to a coulombic efficiency (CE) of 80.1%, which is higher than most reported MoSe_2_ electrodes [14, 24, 40, 41]. The irreversible capacity in the initial cycle may come from the formation of a SEI film [24]. From the second cycle, all charge/discharge curves were close to overlapping, indicating the excellent electrode reversibility.

Figure 3b compares the cycling performances of three samples at 0.1 A g^−1^. MoSe_2_/MoC/N–C nanocomposite exhibits the highest specific capacity of around 800 mAh g^−1^ compared to MoSe_2_/C (650 mAh g^−1^) and bare MoSe_2_ (440 mAh g^−1^). This result demonstrates that constructing interconnected 3D porous carbon-modified nanocomposite can indeed improve the capability of storage Li ion. By the way, in the early stages of the cycling (Fig. 3b), the capacities of MoSe_2_/C and MoSe_2_/MoC/N–C rise slightly, which may be related to the activation of the electrode material. MoSe_2_/MoC/N–C shows the best rate performance (Fig. 3c). The electrode delivered average capacities of 773, 720, 680, 648, 634, 622, and 575 mAh g^−1^ at 0.1, 0.2, 0.5, 1.0, 1.5 2.0, and 4.0 A g^−1^, respectively, and it can fully recover and even displays the higher capacity when the current density switched back to 0.1 A g^−1^. However, MoSe_2_/C and pure MoSe_2_ undergo apparent capacity loss at high rates in different degrees. The obtained high specific capacity and good rate capability of the MoSe_2_/MoC/N–C electrode also show superiority compared to many other carbon-modified MoSe_2_ anode materials (Fig. 3e) [41–47], which demonstrates the advantage of fast interfacial charge transfer through MoSe_2_/MoC/N–C connection again.

What makes the MoSe_2_/MoC/N–C electrode more attractive is its ultralong cycle life with the capacity retention of 90% and a high capacity of 535 mAh g^−1^ after 5000 cycles at 2 A g^−1^ (Fig. 3d). In sharp contrast, for MoSe_2_/C electrode, a capacity of 308 mAh g^−1^ can be maintained just 1000 cycles, and bare MoSe_2_ even undergoes a sharply fading after 295 cycles.

To confirm the role of MoC in lithium storage, three-dimensional porous MoC/C was prepared through a similar routine of preparing MoSe_2_/MoC/N–C. The SEM image (Fig. S8b) shows that MoC/C possesses the similar morphology to MoSe_2_/MoC/C composites (Fig. 2b). The cyclic voltammetry (CV) curves and the cycling performance of MoC/C composite as anode for LIBs were further carried out to study the lithium storage properties of MoC (Fig. S8c, d). As a result, it exhibits the capacity close to that of MoSe_2_/MoC/N–C and good electrochemical reversibility. Meanwhile, the weight ratio between MoSe_2_ and MoC was estimate as 51:2 (molar ratio of 54:5) according to Table S1, indicating that MoC possess relatively low contents in the electrodes. Therefore, the direct capacity contribution from MoC to the whole electrode is relatively limited. However, MoC plays an important role in the interfacial structure regulation, including more stable chemical binding and reduced MoSe_2_ particle size, which is more correlated with the rate performance and structural stability. MoSe_2_/MoC/N–C shows a higher capacity than MoSe_2_/C mainly because its smaller MoSe_2_ nanodots can provide more lithium storage sites. The MoC connected MoSe_2_ and N–C network through C/MoC/MoSe_2_ multiphase boundaries, enabling fast electron/ion transport and structural stability through interface, resulting in good rate performance and structural stability.

Encouraged by the excellent performance MoSe_2_/MoC/N–C electrode in half cell, we assembled a lithium-ion full by pairing with the commercial LiFePO_4_ cathode (Fig. 3f). As shown in Fig. 3g, the full cell displays a complete discharge plateau at about 2.3 V with a reversible capacity of about 267 mAh g^−1^ at 2 A g^−1^. Meanwhile, its energy density was calculated as 93.9 Wh kg^−1^ at the high power density of 698.5 W kg^−1^. It is still a great challenge to obtain stable cycling performance of the full-cell batteries based on conversion reaction electrodes because of the low coulombic efficiency and large consumption of lithium sources during the initial cycles to form SEI layers. However, the LiFePO_4_//MoSe_2_/MoC/N–C full cell exhibits good capacity retention of 86% after 300 cycles (Fig. 3h), indicating the good cyclic stability.

**Fig. 4:**
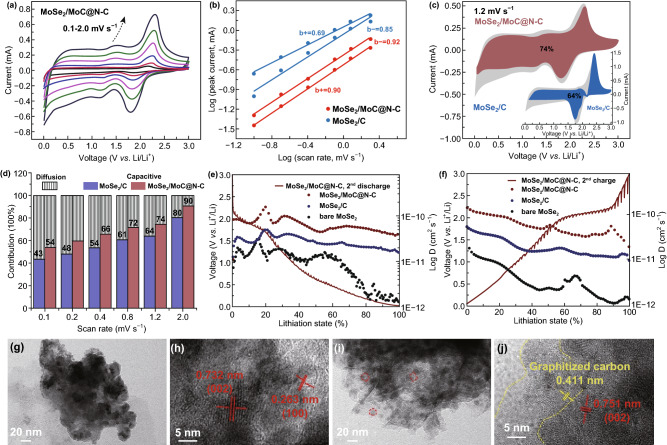
Quantitative capacitive analysis of lithium storage behavior. **a** CV curves at different scan rates of the MoSe_2_/MoC/N–C electrode. **b** Relationship between logarithm cathodic peak current and logarithm scan rates. **c** Capacitive contribution (red wine for MoSe_2_/MoC/N–C and navy blue for MoSe_2_/C) and diffusion contribution (gray) at 1.2 mV s^−1^. **d** Normalized contribution ratio of capacitive capacities at different scan rates; GITT curves and the corresponding Li-ion diffusion coefficient at **e** the 2nd discharge process and **f** the 2nd charge process. TEM and corresponding HRTEM images of **g, h **MoSe_2_/C and **i**, **j** MoSe_2_/MoC/N–C electrodes after 100 cycles at 2 A g^−1^ (full charge state)

The reaction kinetics of MoSe_2_/MoC/N–C electrode was systematically studied by CV measurements, galvanostatic intermittent titration technique (GITT) and EIS spectroscopy. The electrochemical kinetics in the as-prepared electrodes could be considered as capacitive-dominated and diffusion-controlled from the CV curves (Figs. 4a and S9). After detailed calculations as detailed descriptions in the Supporting Information, the higher *b* value of the MoSe_2_/MoC/N–C electrode (0.90 vs. 0.69 of the MoSe_2_/C at the cathodic peak) reveals a faster ionic transportation which resulted in a better rate performance (Fig. 4b) [48–51]. Figure 4c shows the calculated voltage profile for the capacitive current (colored region) at the scan rate of 1.2 mV s^−1^. As a result, 74% of the total capacity is calculated as the capacitive contribution for the MoSe_2_/MoC/N–C electrode, higher than 64% of the MoSe_2_/C. Furthermore, Fig. 4d compares the calculated capacitive contribution of two electrodes at various scan rates. The ratios of capacitive contribution of MoSe_2_/MoC/N–C electrode are always higher than those of MoSe_2_/C, with a maximum value of 90% at 2 mV s^−1^. The large pseudo-capacitive contribution would play a critical role to be benefit for ionic transportation in the electrode [48, 49], which boosting high-rate Li-ion storage capability.

The diffusion-controlled reaction kinetics of samples were further analyzed in depth by GITT (Fig. S11). As shown in Fig. 4e, f, the diffusion coefficient of MoSe_2_/MoC/N–C electrode is about 3.93e−11 to 1.55e−10 cm^2^ s^−1^, higher than those of MoSe_2_/C and bare MoSe_2_, suggesting that expanded layer distance, rich edge defect MoSe_2_ nanocrystal, and MoSe_2_/MoC/N–C connection definitely facilitate fast Li-ion diffusion kinetics. The electrochemical impedance spectra (EIS) results (Fig. S10 and Table S2) demonstrated that charge transfer resistances (*R*_ct_) of MoSe_2_/MoC/N–C (43 Ω) are smaller than those of MoSe_2_/C (72 Ω) and bare MoSe_2_ (557 Ω), suggesting that MoSe_2_/MoC/N–C shows the fastest interface kinetics. All above analysis suggested that the optimized interconnected 3D porous N–C framework and stronger electronic coupling at interface through MoSe_2_/MoC/N–C connection facilitate reaction kinetics.

Ex situ TEM images were employed to investigate the structural integrity of MoSe_2_/MoC/N–C electrode after certain cycles at 2 A g^−1^. Compared to the ex situ TEM images of as-synthesized MoSe_2_/C (Fig. S13A1 and A2), the MoSe_2_ flakes in the MoSe_2_/C electrode (Fig. 4g or Fig. S13A3) were broken into small pieces and form aggregates after 100 cycles, which may further lead to electrode pulverization and indicate its inferior structure stability. However, at the same cycles, MoSe_2_/MoC/N–C electrode (Fig. 4i or Fig S13B3) displayed the good preservation of interconnected 3D porous network structure, and the small nanocrystals encapsulated in the carbon could still be observed, indicating the excellent structural stability of MoSe_2_/MoC/N–C nanocomposite. Compared to the 10-layered structure in Fig. 2k (or Fig. S13B2), the HRTEM image (Fig. 4j or Fig. S13B4) demonstrates that MoSe_2_ nanodots have been exfoliated into less layers (about 4 layers) and smaller size (about 5 nm). The interlayer distance also expanded to 0.751 nm after cycling. In addition, large areas of graphitized carbon were observed with an enlarged spacing (about 0.411 nm), slightly larger than the initial value of 0.370 nm for the pristine carbon. The expanded interlayer distance of MoSe_2_ and carbon could be caused by the repeated insertion/extraction of Li ions [28, 36]. The smaller MoSe_2_ nanocrystal can further provide Li storage sites, benefiting the capacity of electrode. And the increased graphitization carbon layer enhanced the electronic conductivity. These changes of electrodes material during cycling result in climbing capacities. To further examine the reinforced structure stability of MoSe_2_/MoC/N–C, the TEM images of MoSe_2_/MoC/N–C electrode after 500 cycles are compared in Fig. S13B5–B7. Figure S13B5 confirms that the MoSe_2_/MoC/N–C electrode material retains integrated. As shown in Fig. S13B6 and B7, small shadow dots were still distributed in the carbon framework in MoSe_2_/MoC/N–C electrode, implying the products were also evenly constrained in carbon framework. These results prove that MoSe_2_/MoC/N–C connection can constrain the in situ generated MoSe_2_ and facilitate the highly reversible conversion during cycling.

## Conclusion

In conclusion, 3D porous MoSe_2_/MoC/N–C nanocomposite was synthesized by a temperature-induced method. The interface bridging between MoSe_2_ and carbon framework was tuned by MoSe_2_/MoC/N–C connection, greatly improves the structural stability and electronic conductivity and reduces the interfacial charge resistance. Moreover, it also exhibits many features favorable for lithium-ion storage, such as MoSe_2_ nanodots encapsulated into 3D connected carbon network to improve charge/ion transfer kinetics and minimize the effect of volume expansion; expanded interlayer spacing of MoSe_2_ to promote lithium-ion diffusion; large electroactive surface area from N–C and rich edge defects of MoSe_2_; and reinforced structure stability by intermediate in situ MoC. As a result, the as-prepared electrode delivered ultralong and stable cycling performance with a specific capacity of 618 mAh g^−1^ after 5000 cycles (90% capacity retention) at 2 A g^−1^. It also shows promising potential in LiFePO_4_//MoSe_2_/MoC/N–C full cell (86% capacity retention at 2 A g^−1^ after 300 cycles). Inspired by the harvest of superior cell performance, in situ engineering corresponding metal carbide as interphase to connect metal dichalcogenide and carbon matrix can be developed as an innovative and effective strategy to achieve high-electrochemical-activity materials.

## Supporting Information

Experimental section, supplementary scheme diagram of interface modification, TG, XRD, BET, SEM, EIS, CV, GITT and equations associated with this article are given in the online version or from the author.

## Electronic Supplementary Material

Below is the link to the electronic supplementary material.

Supplementary material 1 (PDF 1435 kb)
