# Supplementary material for: Tuning Interface Bridging Between MoSe2 and Three-Dimensional Carbon Framework by Incorporation of MoC Intermediate to Boost Lithium Storage Capability
Source: Nanomicro Lett. 2020 Aug 25;12:171. doi: 10.1007/s40820-020-00511-4 (PMC7770767; doi:10.1007/s40820-020-00511-4)
Supplement: Supplementary file 1 — Supplementary material 1 (PDF 1435 kb) [file 40820_2020_511_MOESM1_ESM.pdf]

Supporting Information for

Tuning Interface Bridging Between $MoSe_2$ and Three-Dimensional Carbon Framework by Incorporation of MoC Intermediate to Boost Lithium Storage Capability

Jing chen[1], Yilin Luo[1], Wenchao Zhang[3], Yu Qiao[2], Xinxin Cao[1], Xuefang Xie[1], Haoshen Zhou[2], Anqiang Pan[1, 4, *], Shuquan Liang[1, *]

[1]School of Materials Science & Engineering, Central South University, Changsha, Hunan 410083, People's Republic of China

[2]Energy Interface Technology Group, National Institute of Advanced Industrial Science and Technology, 1-1-1, Umezono, Tsukuba, 305-8568, Japan

[3]Institute for Superconducting & Electronic Materials, School of Mechanical, Materials, Mechatronics & Biomedical Engineering, Faculty of Engineering and Information Sciences, University of Wollongong, NSW 2500, Australia

[4]Key Laboratory of Advanced Energy Materials Chemistry (Ministry of Education), Nankai University, Tianjin 300071, People's Republic of China

*Corresponding authors. E-mail: pananqiang@csu.edu.cn (Anqiang Pan); lsq@csu.edu.cn (Shuquan Liang)

Supplementary Figures and Tables

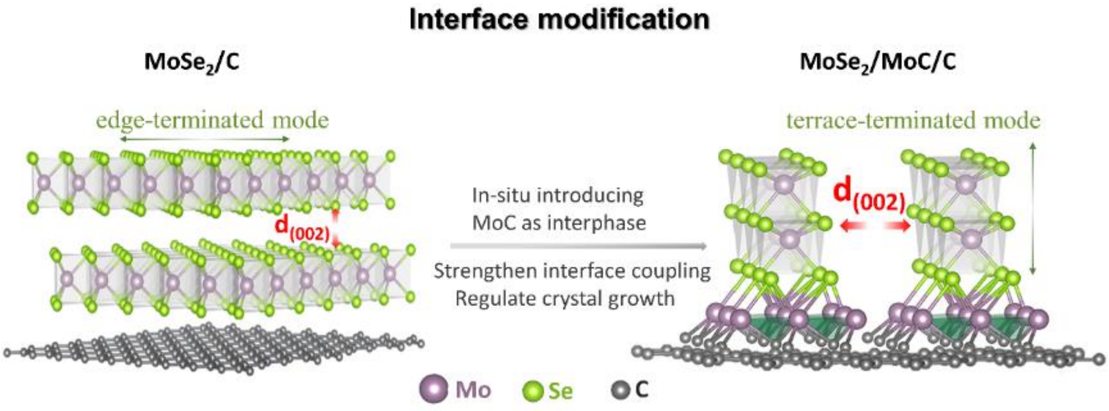

Scheme S1 Schematic illustration of models of $MoSe_2$/C and $MoSe_2$/MoC/C heterostructure

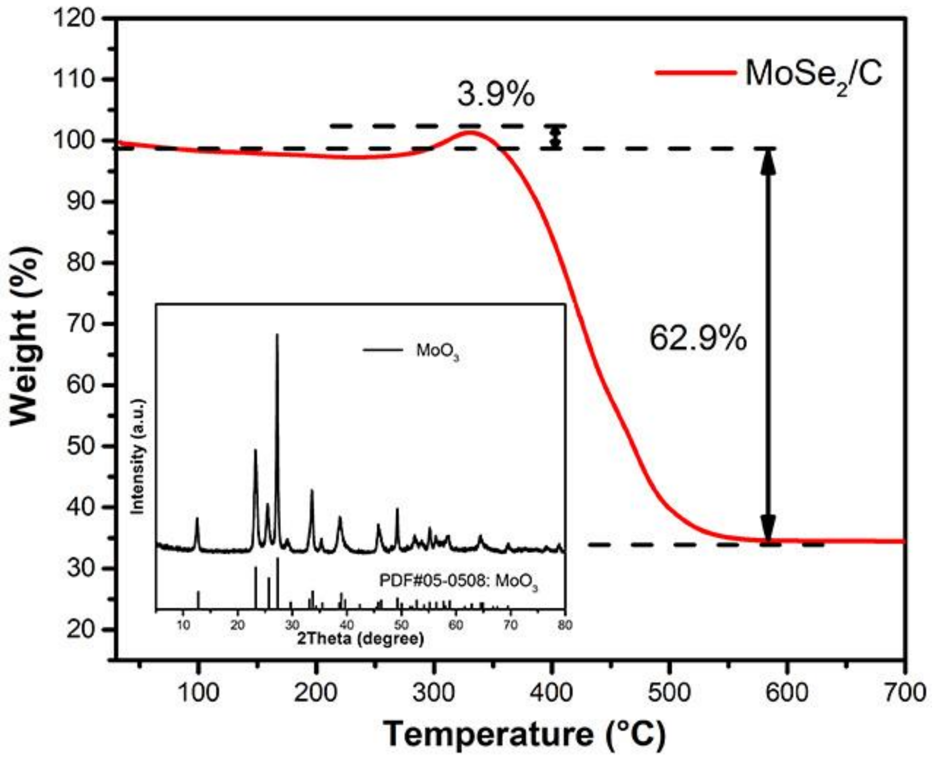

Fig. S1 TG curve of $MoSe_2$/C from room temperature to 700 °C at a heating rate of 10°C min^{-1}, the inset shows the XRD pattern of products after the TG test

During TG test, $MoSe_2$ oxidized to MoO_3 and SeO_2. And the reduced part is related to the leaving of SeO_2 and CO_2:

$$2MoSe_2 + (x+7)O_2 + xC = 2MoO_3 + 4SeO_2 + xCO_2 \quad \textbf{(S1)}$$

As a result, the carbon content of $MoSe_2$/C composite could be calculated as 34.6%.

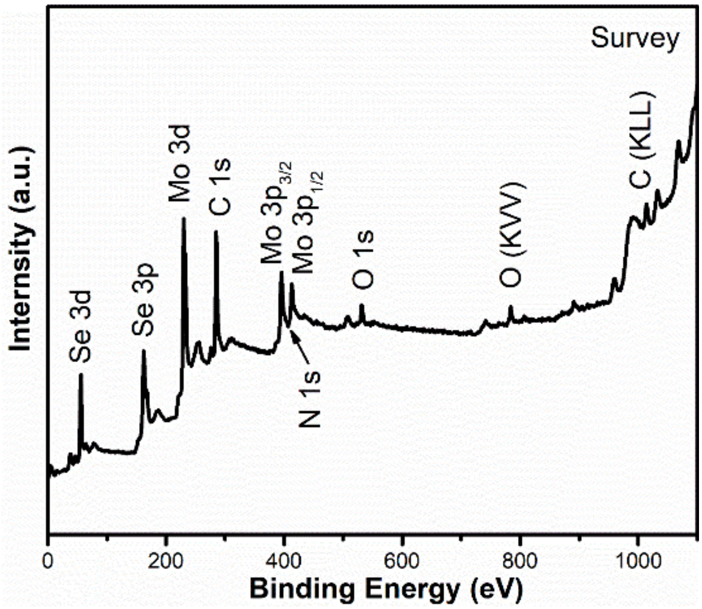

Fig. S2 XPS survey spectrum of $MoSe_2$/MoC@N-C sample

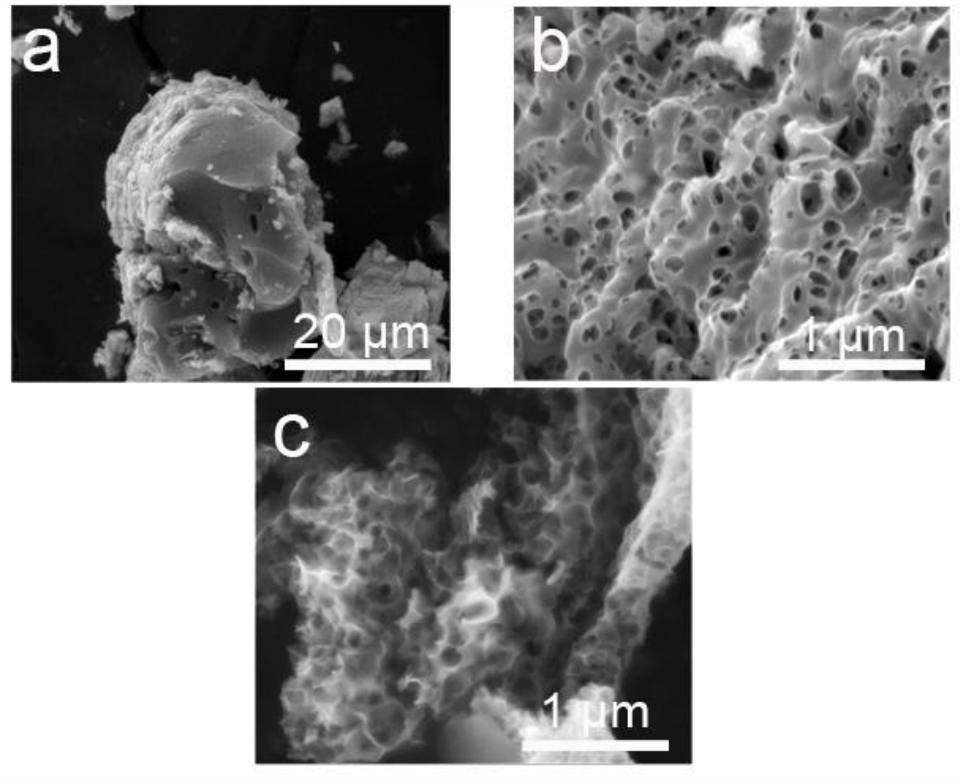

Fig. S3 SEM images (**a**) bare $MoSe_2$, (**b**) $MoSe_2$/C, and (**c**) $MoSe_2$/MoC@N-C

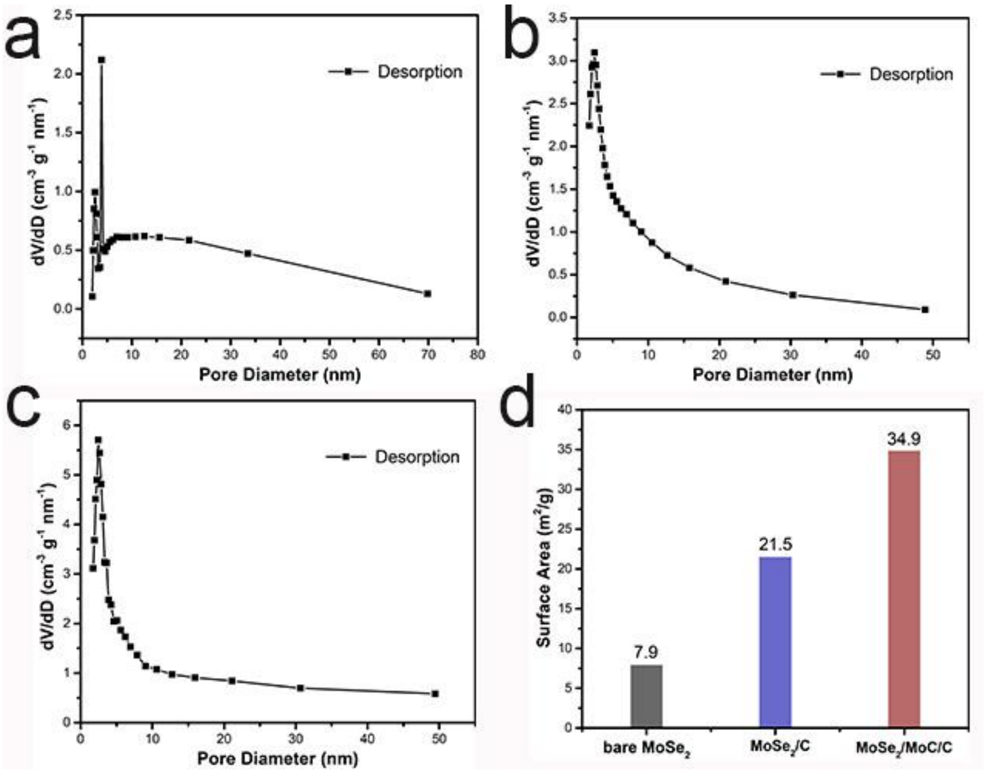

Fig. S4 Pore size distribution of three samples: (**a**) bare $MoSe_2$, (**b**) $MoSe_2$/C, (**c**) $MoSe_2$/MoC/C; (**d**) statistical figure of specific surface area of these three sample

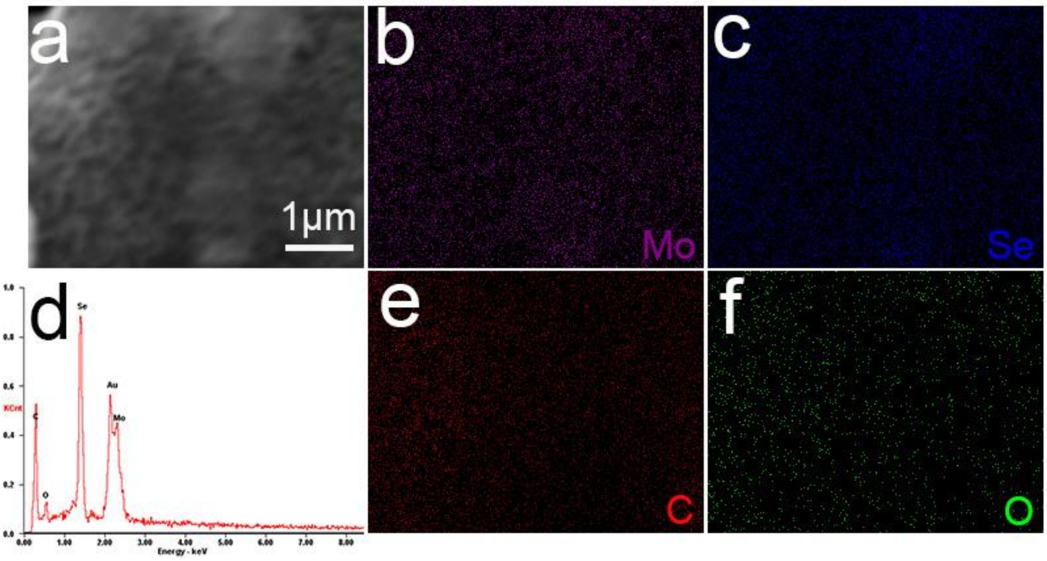

Fig. S5 EDS mapping of $MoSe_2$/MoC/N-C sample

Table S1 Elemental composition of $MoSe_2$/MoC/N-C from EDS mapping results

Elements	Atomic %	wt%
Se	4.87	17.66
Mo	2.66	11.73
C	83.4	45.92
O	6.9	5.06
Au	2.17	19.63

According to the elemental analysis result, the atomic ratio of Se to Mo is 1.831 in $MoSe_2$/MoC/N-C composite. For $MoSe_2$, the atomic ratio of Se to Mo is 2:1. The excess Mo can be attributed to MoC. Therefore, the molar ratio of $MoSe_2$ and MoC can be estimated as 54:5, and the mass ratio was calculated as 51:2.

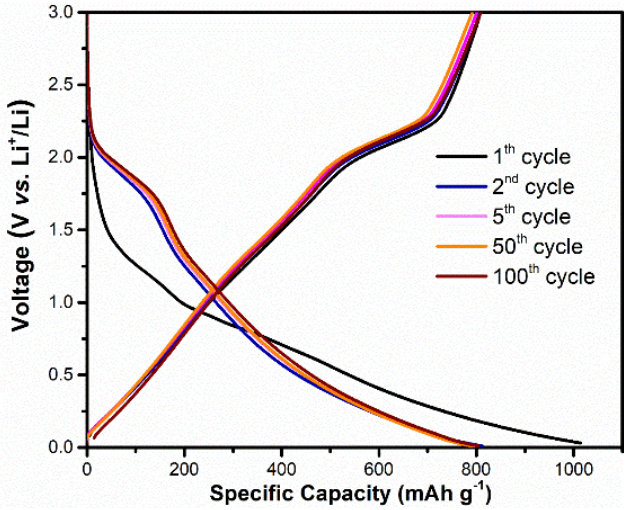

Fig. S6 Charge/discharge profiles of $MoSe_2$/MoC@N-C electrode at 0.1 A g^{-1}

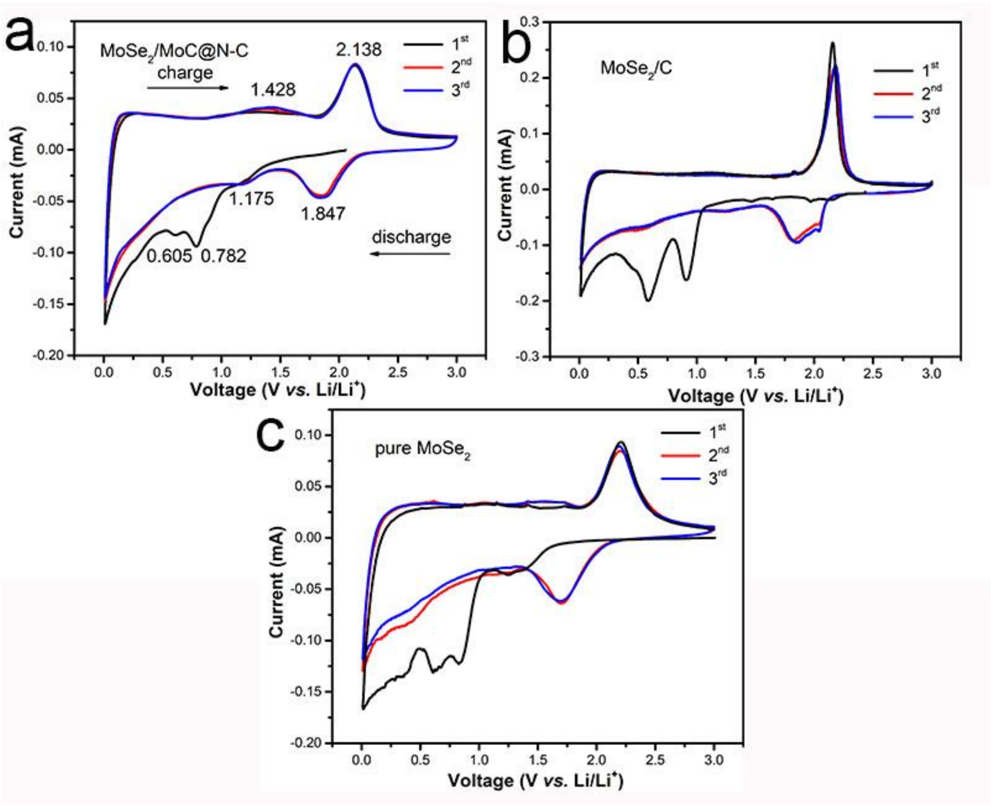

Fig. S7 First to third cyclic voltammograms of electrodes at a scan rate of 0.1 mV S^{-1} of (**a**) $MoSe_2$/MoC@N-C, (**b**) $MoSe_2$/C, and (**c**) bare $MoSe_2$

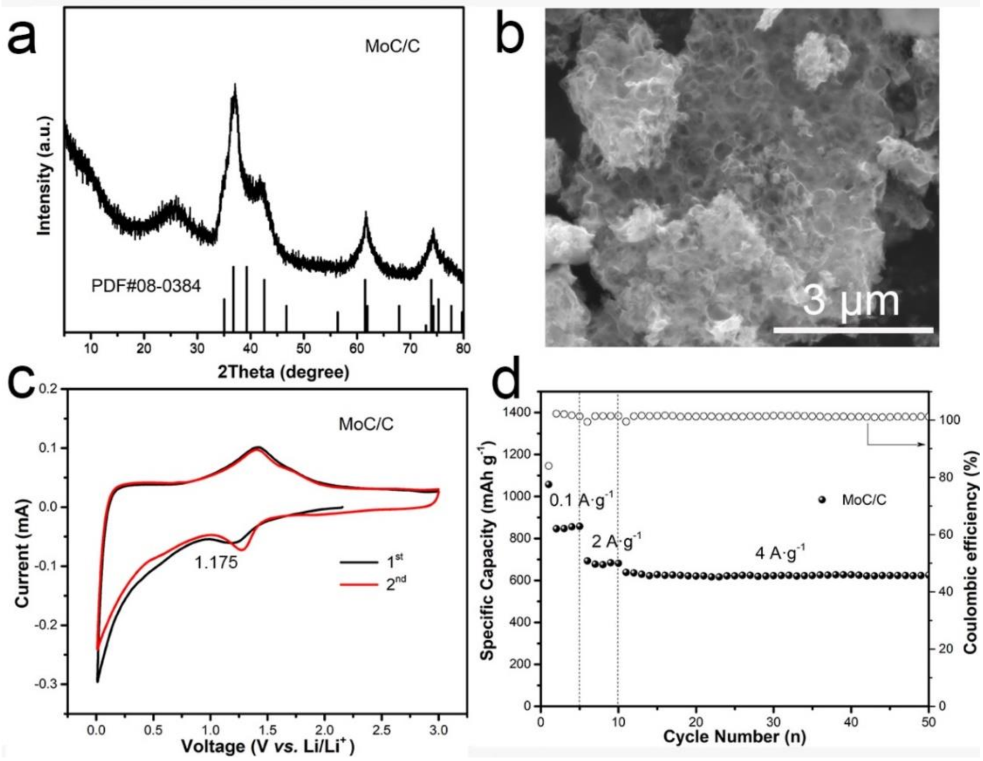

Fig. S8 XRD data (**a**) and SEM image (**b**) of MoC/C sample, (**c**) CV curves and (**d**) cycling performance of MoC/C electrode as anode of LIBs

Three dimensional porous MoC/C was synthesized by the similar method without Se powder. As shown in **Fig. S8a**, the XRD patterns exhibited several peaks matched well with MoC phase. The scanning electron microscopic (SEM) image (**Fig. S8b**) show that the morphologies of MoC/C was similar to the as-synthesized $MoSe_2$/MoC/C composites (**Fig. 2b**). To investigate the performance of MoC/C as anode for LIBs, CV and cycling test were conducted. As shown in **Fig. S8c**, the small peak at 1.175 V can be assigned to the reduction of MoC, and this peak almost remains constant in the second cycle, suggesting the reversible conversion reactions of MoC. These results are in good agree with that of CV curves of $MoSe_2$/MoC/N-C in **Fig. 3a**. **Figure S8d** shows the cycling performances of MoC/C sample at various current densities. MoC/C nanocomposite exhibits specific capacities of around 820, 640 and 590 mAh g^{-1} at 0.1, 2.0 and 4.0 A g^{-1}, respectively, comparable to those of $MoSe_2$/MoC/N-C electrode (773, 622 and 575 mAh g^{-1} at 0.1, 2.0 and 4.0 A g^{-1}, respectively).

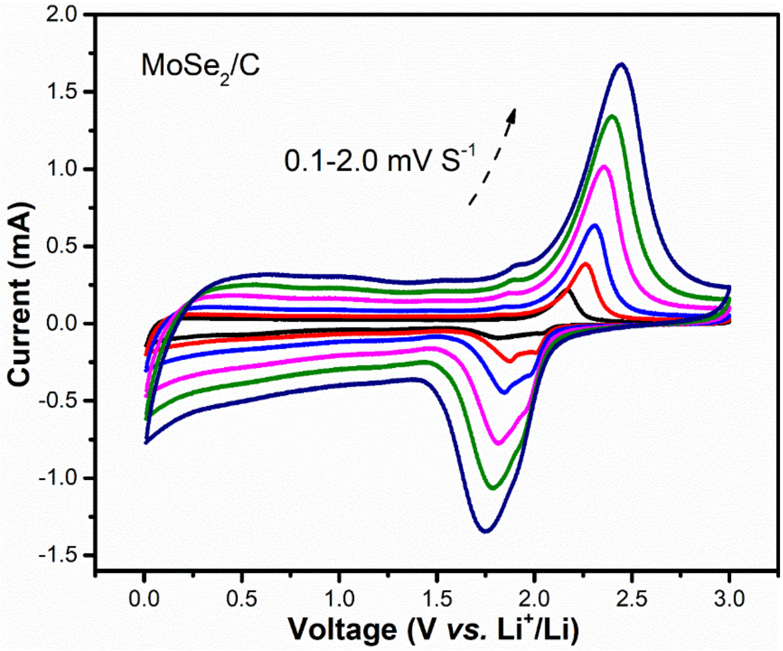

Fig. S9 CV curves at different scan rates of the $MoSe_2$/C electrode

The degree of capacitive effect can be qualitatively analyzed according to the relationship between measured current (i) and scan rate (v) from the CV curves [S1, S2]:

$$i = av^b = k_1 v + k_2 v^{1/2} \qquad \text{(S2)}$$

where *a* and *b* both are constants, *v* is the sweep rate (mV S^{-1}) and *i* (V) is the current (mA) at the corresponding sweep rate. The value of *b* is between 0.5 and 1.0, which is determined from the slope of the log *i versus* log *v* plot. It is well known that for a surface capacitance-dominated process *b* is close to 1.0, while a b-value of 0.5 signifies a solid-state diffusion-controlled reaction.

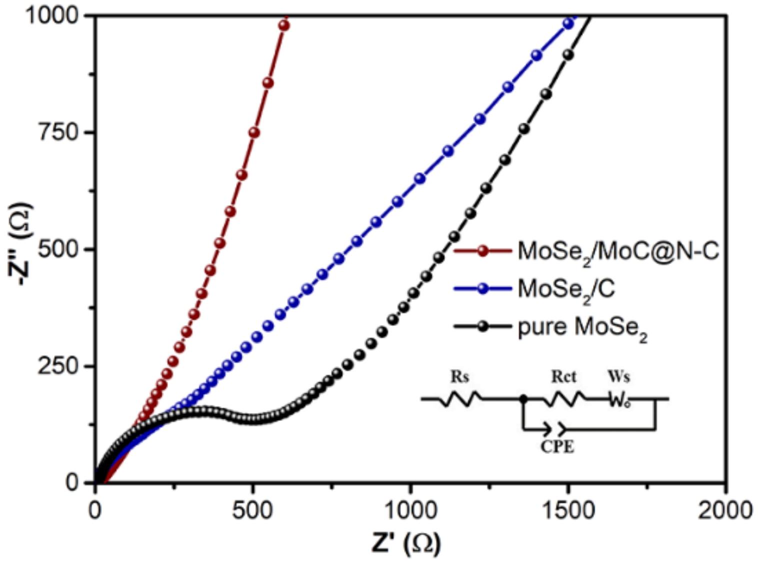

Fig. S10 Nyquist plots of pure $MoSe_2$, $MoSe_2$/C and $MoSe_2$/MoC@N-C electrodes after 1 cycle at 0.1 A g^{-1}, the inset shows the equivalent circuit. The semicircle in the medium-frequency band is related to the electrochemical reaction impedance, while the inclined line in the low frequency zone is related to the solid-state ion diffusion. The spectra are fitted by a classic equivalent circuit (the inset of **Fig. S10**), where Rct is the charge-transfer resistance at the interfaces and Zw is the Warburg impedance related to Li^+ diffusions [S3].

Table S2 Fitting results of Rct in equivalent circuit for Nyquist plots of **Fig. S8**

Samples	Bare $MoSe_2$	$MoSe_2$/N-C	$MoSe_2$/MoC/N-C
Rct (Ω)	557	72	43

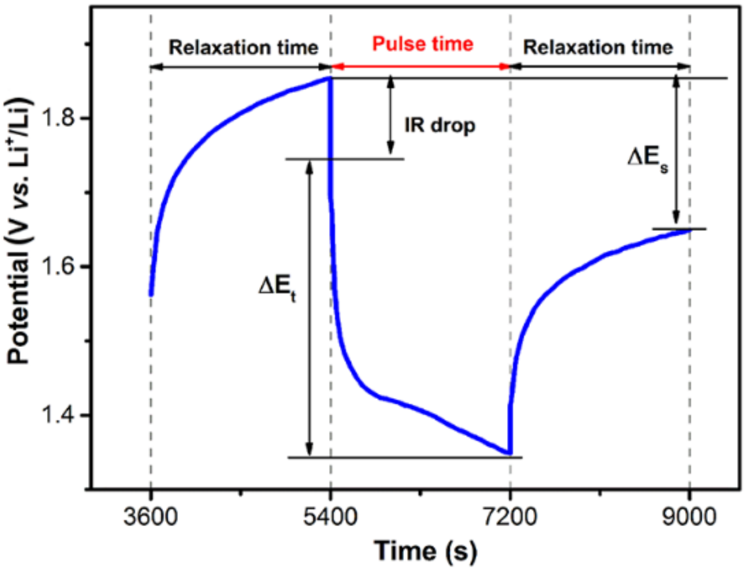

Fig. S11 E *vs.* t curves of $MoSe_2$/MoC@N-C electrode for a single GITT during discharge process

The lithium diffusion coefficient was measured by using Galvanostatic Intermittent Titration Technique (GITT) and calculated based on **Eq. S3** as follows [S3]:

$$D = \frac{4L^2}{\pi\tau}\left(\frac{\Delta Es}{\Delta Et}\right)^2 \qquad \textbf{(S3)}$$

Where t is the duration of the current pulse (s), τ is the relaxation time (s), and ΔEs is the steady-state potential change (V) by the current pulse. ΔEt is the potential change (V) during the constant current pulse after eliminating the iR drop. L is lithium ion diffusion length (cm); for compact electrode, it is equal to thickness of electrode.

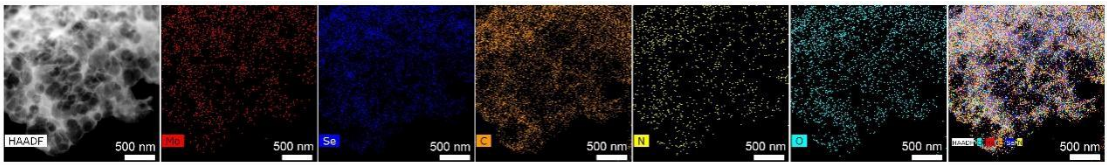

Fig. S12 Elemental mappings of $MoSe_2$/MoC/N-C

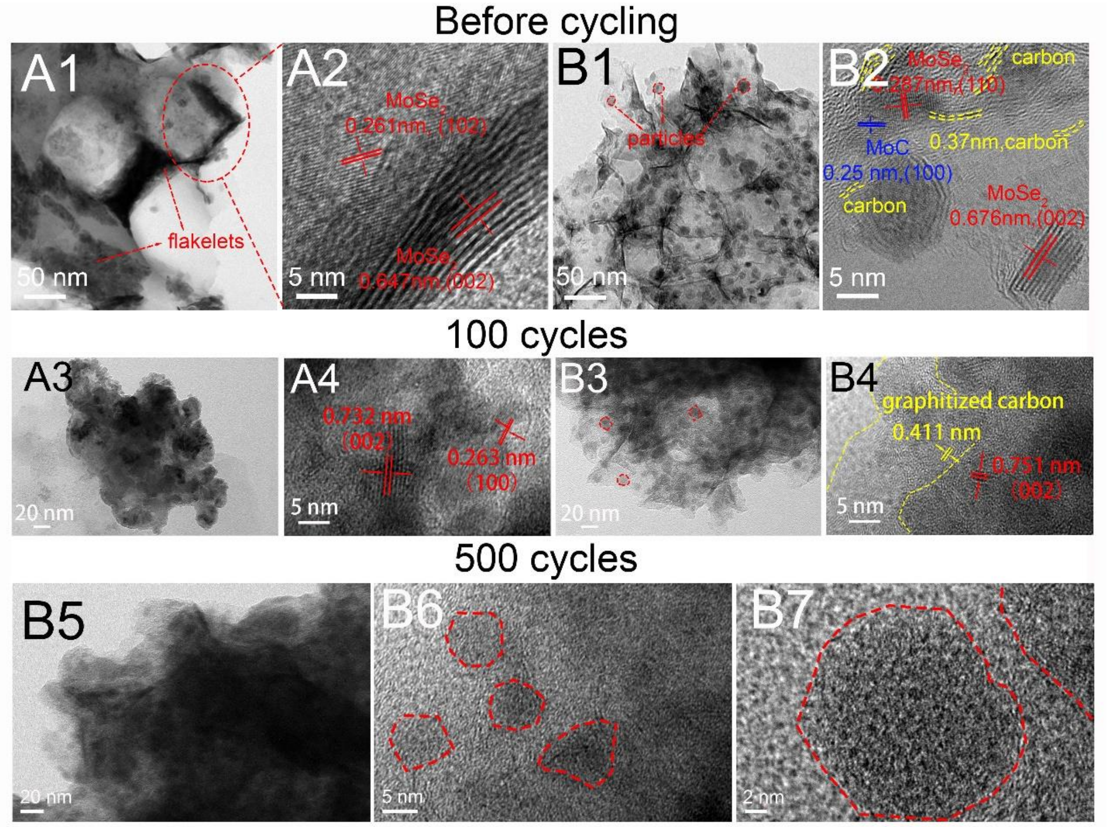

Fig. S13 *Ex-situ* TEM images of as prepared samples and electrodes after cycled at 2 A g^{-1}: (**A1-A4**) $MoSe_2$/N-C sample and corresponding electrode, (**B1-B7**) $MoSe_2$/MoC/N-C sample and corresponding electrode

Supplementary References

[S1]D. L. Chao, P. Liang, Z. Chen, L. Y. Bai, H. Shen et al., Pseudocapacitive Na-ion storage boosts high rate and areal capacity of self-branched 2d layered metal

chalcogenide nanoarrays. ACS Nano **10**, 10211-10219 (2016). https://doi.org/10.1021/acsnano.6b05566

[S2]D. L. Chao, C. R. Zhu, P. H. Yang, X. H. Xia, J. L. Liu et al., Array of nanosheets render ultrafast and high-capacity Na-ion storage by tunable pseudocapacitance. Nat. Commun. **7**, 12122 (2016). https://doi.org/10.1038/ncomms12122

[S3]J. Chen, A. Pan, Y. Wang, X. Cao, W. Zhang et al., Hierarchical mesoporous $MoSe_2$@cose/n-doped carbon nanocomposite for sodium ion batteries and hydrogen evolution reaction applications. Energy Storage Mater. **21**, 97-106 (2018). https://doi.org/https://doi.org/10.1016/j.ensm.2018.10.019
